# Plant quality and local adaptation undermine relocation in a bog specialist butterfly

**DOI:** 10.1002/ece3.427

**Published:** 2012-12-26

**Authors:** Camille Turlure, Viktoriia Radchuk, Michel Baguette, Mark Meijrink, Arnold den Burg, Michiel Wallis Vries, Gert-Jan Duinen

**Affiliations:** 1 Earth and Life Institute, Universite catholique de Louvain – Biodiversity Research CentrePlace Croix du Sud, 4, 1348, Louvain-la-Neuve, Belgium; 2 Muséum National d‘Histoire Naturelle (MNHN) - Ecologie et Gestion de la Biodiversité, Avenue du Petit Château 1, 91800 BrunoyFrance; 3 CNRS USR 2936, Station d‘écologie expérimentale du CNRS09200 Moulis, France; 4 University of Applied Sciences Van Hall LarensteinVelp, The Netherlands; 5 Bargerveen Foundation / Radboud University Nijmegen, Institute of Water and Wetland Research, Department of Animal EcologyNijmegen, The Netherlands; 6 Dutch Butterfly Conservation, De VlinderstichtingWageningen, The Netherlands; 7 Wageningen University, Laboratory of EntomologyWageningen, The Netherlands

**Keywords:** Applied ecology, conservation, evolutionary ecology, insect-plant interactions, local adaptation, relocation

## Abstract

The butterfly *Boloria aquilonaris* is a specialist of oligotrophic ecosystems. Population viability analysis predicted the species to be stable in Belgium and to collapse in the Netherlands with reduced host plant quality expected to drive species decline in the latter. We tested this hypothesis by rearing *B. aquilonaris* caterpillars from Belgian and Dutch sites on host plants (the cranberry, *Vaccinium oxycoccos*). Dutch plant quality was lower than Belgian one conferring lower caterpillar growth rate and survival. Reintroduction and/or supplementation may be necessary to ensure the viability of the species in the Netherlands, but some traits may have been selected solely in Dutch caterpillars to cope with gradual changes in host plant quality. To test this hypothesis, the performance of Belgian and Dutch caterpillars fed with plants from both countries were compared. Dutch caterpillars performed well on both plant qualities, whereas Belgian caterpillars could not switch to lower quality plants. This can be considered as an environmentally induced plastic response of caterpillars and/or a local adaptation to plant quality, which precludes the use of Belgian individuals as a unique solution for strengthening Dutch populations. More generally, these results stress that the relevance of local adaptation in selecting source populations for relocation may be as important as restoring habitat quality.

## Introduction

Many of the evaluated species of different taxonomic groups are currently considered endangered, their decline being closely linked to the multiple negative impacts of human activities (including habitat degradation and fragmentation, introduction of invasive species and pollution; Millenium Ecosystem Assessment 2005). However, extinction risks may even be underestimated as studies often ignore species losses through co-extinction of interacting species (Diamond 1989). The study by Koh et al. (2004) provides a clear example. They modeled the status of affiliate species with host species currently listed as endangered, and estimated that 200 species went extinct due to the extinction of their host species, and more than 6000 species need to be considered as co-endangered. Given that complex interactions among species are most often only poorly documented (Moir et al. 2010; Colwell et al. 2012), preservation of every single species as a potential host for several others is of prime importance. This is particularly an issue for specialist species, which are overrepresented in the lists of species of conservation concern (e.g. Kuussaari et al. 2009).

Population persistence of habitat specialists is conditioned by several factors, such as connectivity with other local populations within a meta-population or availability of a sufficient area and quality of habitat (e.g. Hanski 1999; Thomas et al. 2001). In butterflies, habitat quality depends on the distribution, amount, and quality of the different resources needed by each of the four developmental stages (i.e. eggs, caterpillars, pupae, and adults; see the definition of habitat based on resources developed by Dennis et al. 2003). There is accumulating evidence that host plant quality is essential for caterpillars to achieve optimal growth and survival (Scriber 2010). Nitrogen has been recognized as a critical limiting nutrient for the organisms (Mattson 1980) and was shown to be beneficial for larval development, enhancing growth rate, and survival in several species (see Throop and Lerdau 2004 for a review; Hwang et al. 2008 for an example with *Pieris* butterflies). However, this may not apply to a specialist species in formerly oligotrophic environments that became highly enriched by increased atmospheric nitrogen deposition over the last decades.

At the community level, anthropogenic nitrogen input has led to declines in plant diversity (Weiss 1999; Bobbink et al. 2003; Stevens et al. 2004) and may consequently be detrimental to insect communities (see Ockinger et al. 2006; WallisDeVries and Van Swaay 2006). At the population level, several factors may explain the decline in specialist butterflies under high nitrogen input. An increase in nutrient availability and hence plant productivity leads to habitat loss for habitat specialists (Oostermeijer and Van Swaay 1998), in particular as a result of decreasing host plant abundance and a deterioration of microclimatic conditions (WallisDeVries and Van Swaay 2006). An alternative explanation, proposed by van den Burg (2006), is the reduction in quality of larval food plants through alterations in the nutritional balance as a result of increased nitrogen levels. This explanation is still mainly hypothetical, but there is experimental evidence of negative effects of excess nitrogen on larval development in the herbivorous butterfly *Lycaena tytirus* from oligotrophic ecosystems (Fischer and Fiedler 2000). Similarly, Nijssen and Siepel (2010) found a decline in the body weight of the marbled grasshopper *Myrmeleotettix maculata* with increasing nitrogen content in the grass *Corynephorus canescens* in inland drift sands in The Netherlands under different levels of atmospheric nitrogen deposition. Moreover, the anthropogenic nitrogen input can differ greatly from one region to another, leading to divergent adaptation of the populations occurring in these regions (reviewed by Scriber and Slansky 1981 and Scriber 2010).

Relocation of individuals (i.e. “any intentional movement by humans of an animal or a population of animals from one location to another”; Fischer and Lindenmayer 2000) is one of the several options to prevent regional species extinction. It includes introduction (the establishment of “a species outside its recorded distribution”), reintroduction (the establishment of “a species in an area which was once part of its historical range”), translocation (the “movement of wild individuals or populations from one part of their range to another”) and supplementation (the addition of individuals to an existing population). The last three relocation types are especially appropriate when local populations have drastically decreased in size, went extinct or when spontaneous recolonization is not likely, even after habitat restoration (Richardson et al. 2009). Attempts at relocations have been made successfully for different kinds of organisms (see e.g. *Equus hemionus* in Saltz and Rubenstein 1995; *Gyps fulvus* in Sarrazin and Legendre 2000; *Petroica australis* in Armstrong and Ewen 2002; Maschinski and Duquesnel 2007), including butterflies (see references below).

Kleiman (1989) and Armstrong and Seddon (2008) identified prerequisites for successful relocation attempts: (1) the need for sufficient habitat quality in the release area, (2) the elimination of factors causing species decline, (3) the knowledge of the species requirements and behavior, and (4) the training of individuals before release. Improvement of habitat quality prior to relocation was given consideration previously in butterflies (*Maculinea arion*: Elmes and Thomas 1992; *Pseudophilotes baton schiffermuelleri*: Marttila et al. 1997; butterfly community: Waltz & Covington, 2004). If the first three prerequisites are met, relocations are typically applied, or tested via Population Viability Analysis (Morris and Doak 2002), under the assumption that relocated individuals perform equally well as if they would do on their native site. Nevertheless, several studies have shown that this assumption may be violated (Fischer and Lindenmayer 2000; Stamps and Swaisgood 2007). This is especially true in the case of releasing captive-bred animals (Seddon et al. 2007). For example, a recent study on the grey partridge revealed a maladaptive habitat preference of released individuals, leading to lower survival rate and hence fitness (Rantanen et al. 2010). In the UK, several reintroductions of the butterfly *Lycaena dispar* have been attempted, but have ultimately failed, probably because butterflies from a Dutch breeding stock of another subspecies were not locally adapted to the habitat conditions at the release site (Nicholls and Pullin 2000). Therefore, the performance of individuals should be tested prior to their relocation using appropriate experimental design and/or followed-up by long-term monitoring of the populations (Sarrazin and Barbault 1996; Seddon et al. 2007). Indeed, as stated by Sarrazin and Barbault (1996), relocated individuals may “lack locally selected traits that are likely to have existed in the extinct population” (p. 475).

**Figure 1:**
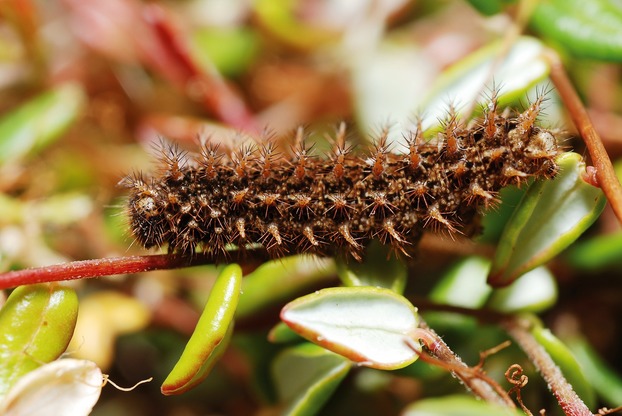
Picture of *Boloria aquilonaris* caterpillar in its habitat (by Gilles San Martin).

The cranberry fritillary butterfly, *Boloria aquilonaris* (Stichel 1908), is an oligotrophic bog specialist species of conservation concern in Western Europe. Previous studies revealed that the habitat of this species consists of (1) *Sphagnum* hummocks covered by the host plant, *Vaccinium oxycoccos*, providing suitable resources and micro-environmental conditions for the caterpillars (Fig. 1; Turlure et al. 2010a,b) and (2) various nectar feeding resources for the adults (Turlure et al. 2010c). Viability of different meta-populations of *B. aquilonaris* has been estimated in two different countries and predicted stable metapopulation dynamics in Belgium versus metapopulation collapse in The Netherlands (Schtickzelle et al. 2005). From a conservationist's view, this situation could pave the way for reintroduction and/or supplementation (Hoegh-Guldberg et al. 2008) to rescue the collapsing Dutch metapopulation. Reintroduction and supplementation of *B. aquilonaris* individuals could be necessary to establish populations in potentially suitable but unoccupied sites (van Swaay and WallisDeVries 2001) and restore declining populations in the Netherlands, respectively. Indeed, several recent reintroduction trials for butterflies have proven to be successful and resulting in population establishment (*Maculinea teleius* and *M. nausithous* in Wynhoff 1998; *M. arion* in Thomas et al. 2011; *Pseudophilotes baton schiffermuelleri* in Marttila et al. 1997), justifying an attempt with *B. aquilonaris*.

The aim of this study was thus to test whether the relocation of *B. aquilonaris* individuals from a large Belgian metapopulation (estimated at around 9000 and 10,000 individuals in 2010 and 2011, respectively; C. Turlure, unpubl. data) to the Dutch populations is a reasonable conservation strategy. With this goal in mind, we tested two conditions that should be fulfilled for a reintroduction and/or supplementation to be successful:

1. Sufficiently high habitat quality of the Dutch receiver sites to support relocated individuals. A decrease in host plant quality (i.e. imbalance due to increased atmospheric nitrogen deposition or the absence of minerotrophic ground water within the root zone of *V. oxycoccos*) as a driver for species decline in the Netherlands has not been investigated yet, but effects of increasing nitrogen deposition on nutrient ratios in vascular plants and *Sphagnum* mosses have been found in ombrotrophic bogs (Tomassen et al. 2004; Jirousek et al. 2011). Therefore, the performance of *B. aquilonaris* caterpillars fed with *V. oxycoccos* plants from the two different countries was tested. If, as expected, the quality of the host plant is lower in the Netherlands, we should observe a decrease in growth rate and in survival for caterpillars fed with Dutch plants. Alternatively, if plant quality is similar, similar growth rate and survival between groups of caterpillars fed with the plants from the two different origins should be observed.
2. A similar performance of native individuals and relocated individuals from the source population. As mentioned above, reintroduction and/or supplementation may be necessary to ensure the viability of the cranberry fritillary in the Netherlands. The caterpillar stage would be the most effective stage to relocate individuals because eggs and pupae are very vulnerable to manipulation (C. Turlure & V. Radchuk, pers. obs.) and manipulations of adults may influence their behavior at the release site, as has been observed for other species (Heidinger et al. 2009). To test this hypothesis, the performance of Belgian and Dutch caterpillars fed with plants from the two different countries were compared. If some traits were selected in caterpillars to cope with the gradual change in host plant quality during the last decades, caterpillars should perform better when fed with food from their country of origin. Depending on the strength of the relationship, local adaptation to host plant quality could preclude the use of the Belgian population as a source population for reintroduction and/or supplementation in Dutch sites.

Results of the two experiments have a strong applied value, in determining the conservation measures for this vulnerable species, especially in the Netherlands. More generally, our experiments will provide insights into how local environments may influence relocation success.

## Methods

### Study species

The cranberry fritillary, *B. aquilonaris*, is a glacial relict butterfly of acid peat bogs and damp heaths. Adults fly in July and females lay their eggs singly on the underside of the leaves of the host plant, *V. oxycoccos*. This species has a boreo-alpine distribution and is listed as vulnerable in the Red Data Book of European butterflies (van Swaay and Warren 2006).

### Caterpillar rearing

In May 2010, 76 caterpillars were collected from the Fange de Crépale (50°16′N 5°44′E; elevation: 565 m) and the Grande Fange (50°14′N 5°46′E; elevation: 555 m) peat bog reserves in Belgium and 18 caterpillars from the Schoonloo peat bog (52°53′N 6°42′E; elevation: 24 m) in the Netherlands. Caterpillars were assigned to one of two size groups (caterpillars at the final instar and caterpillars at the penultimate instar). They were kept individually in Petri dishes provided with a piece of wet cotton and ad libitum access to young shoots of the host plant, and placed in a climate room (light from 0800 to 2000 h with 18°C vs. dropping the temperature to 10°C under dark conditions to mimic natural temperature and light fluctuations). Every 2 or 3 days, each caterpillar was (1) weighed using a precise balance (Mettler Toledo MT5; resolution: 0.01 mg, precision: 0.02 mg) and (2) its Petri dish was cleaned and provided with a clean piece of wet cotton and fresh young host plant leaves. The food plant shoots used to feed the caterpillars were collected several times a week in the Grande Fange and the Schoonloo peat bogs in Belgium and the Netherlands respectively, and stored at 4°C for a maximum of 3 days before use. Emerging butterflies were released at the site from which they were collected as a caterpillar.

The experiment was split into two parts. In the first part, the 18 Dutch caterpillars and 21 of the Belgian caterpillars were used to test the effect of food and caterpillar origin (Belgian vs. Dutch) in a complete factorial design (later referred to as Experiment I). In a second part, the 55 additional Belgian caterpillars were used to test the effect of food origin and caterpillar instar (penultimate vs. final instar) (later referred to as Experiment II). Each caterpillar was randomly assigned to one of the two food treatments.

### Data analysis

#### Growth rate

Most caterpillars did not increase their weight following 2 weeks after the start of the experiment, either due to entering pupation (indeed, caterpillars are losing weight before pupation) or for some other caterpillars, unknown reasons. Therefore, in order to keep all the individual data for the analysis, growth rate of each *B. aquilonaris* individual was calculated as weight on the 3rd, 5th, 7th, and 10th day (i.e. a few days before the first caterpillar pupation) divided by initial weight. In Experiment I, we tested the effect of time, food origin (i.e. Belgian vs. Dutch food), caterpillar origin, and interaction between food origin and caterpillar origin on caterpillar growth rate using generalized linear regression. In Experiment II, we tested the effect of time, food origin (i.e. Belgian vs. Dutch food), caterpillar instar at the beginning of the rearing (final vs. penultimate as described above), and interaction between caterpillar instar and food origin on caterpillar growth rate using generalized linear regressions (Proc Genmod in SAS; SAS Institute Inc. 2003; Anderson 2008). For regression models, the statistical approach was to fit models corresponding to all possible combinations of the factors (i.e. nine models) and select the best model with the smaller AICc value and the lowest number of parameters (see details in Burnham and Anderson 2002; Anderson 2008).

#### Survival

We defined the individuals reaching the (pre-)pupae stage as survivors. We tested the effects (1) of food and caterpillar origin on caterpillar survival from Experiment I and (2) of food origin and caterpillar instar at the start of the experiment on caterpillar survival from Experiment II using logistic regression models and appropriate contrasts (Proc Logistic in SAS; SAS Institute Inc. 2003; Anderson 2008).

## Results

### Experiment I: effects of plant and caterpillar origins

Most (72%) of the Dutch caterpillars were at the last instar stage at the beginning of the experiment, whereas most (67%) of the Belgian caterpillars were at the penultimate instar. Dutch caterpillars were therefore initially on average heavier than Belgian ones (Caterpillar origin: *F*_1,38_ = 6.32, *P* = 0.017; mean weight of Dutch caterpillars = 76.99 ± 20.14 mg; mean weight of Belgian caterpillars = 49.71 ± 11.26 mg), but there was no difference in initial weight between the two food treatments nor interaction effect (Food origin: *F*_1,38_ = 0.03, *P* = 0.87; Interaction: *F*_1,38_ = 0.001, *P* = 0.97).

**Figure 2:**
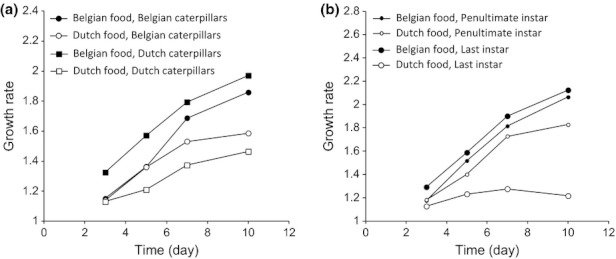
Changes in caterpillar mean growth rate in time according to (a) the food origin (Black symbols: Belgian food; White symbols: Dutch food) and caterpillar origin (circles: Belgian caterpillars; squares: Dutch caterpillars) and (b) caterpillar instar at the beginning of breeding (Small symbols: penultimate instar; Large symbols: last instar) and food treatment (Black circles: Belgian food; White circles: Dutch food).

Growth rate was affected by time, caterpillar's origin, food origin and interaction between food origin and caterpillar origin (see Appendix 1a for model selection and Table 1 for parameter estimates using the best model). Caterpillar growth rate increased over time as expected and was on average higher for Belgian caterpillars and for caterpillars fed with Belgian plants. Additionally, growth rate was increasing more slowly for caterpillars of both countries fed with Dutch plants (Fig. 2a).

**Table 1:** Factors affecting caterpillar growth rate (estimated using best model from Appendix 1a)

Parameter	Level	Estimate	Std
Intercept		0.8059	0.0791
Time		0.0782	0.0094
Caterpillar origin	Belgium	0.1103	0.0696
Food origin	Belgium	0.3713	0.0728
Caterpillar origin*Food origin	Belgium*Belgium	−0.2605	0.0979

For categorical variables, the estimate expresses the difference of the presented level with the reference level (fixed to zero). Caterpillar growth rate (1) increased with time, (2) was on average higher for Belgian caterpillars, (3) was on average higher for caterpillars fed with Belgian plants, and (4) was increasing more slowly for caterpillars of both countries fed with Dutch plants.

**Figure 3:**
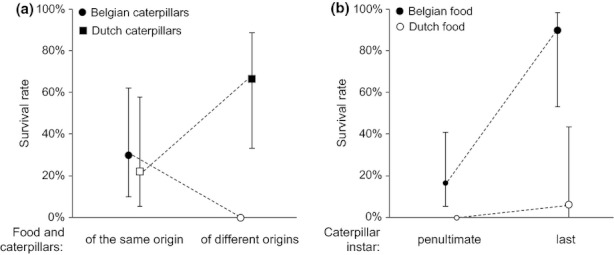
Estimated survival rate (±95% confidence interval) of caterpillars under the different rearing conditions: (a) according to origin (circles: Belgian caterpillars; squares: Dutch caterpillars) and food treatment (Black symbols: Belgian food; White symbols: Dutch food; Experiment I), (b) for Belgian caterpillars only according to food treatment (Black symbols: Belgian food; White symbols: Dutch food) and initial instar (Small symbols: penultimate instar; Large symbols: last instar; Experiment II).

Among the 39 caterpillars, 28% reached the (pre-)pupae stage and 72% died. Results of the logistic regression indicated a significant effect of food origin (χ^2^ = 8.64; *P* = 0.0033) and caterpillar origin (χ^2^ = 5.83; *P* = 0.0158) on the survival rate. On average, higher survival rates were observed (1) for caterpillars fed with Belgian plants compared with caterpillars fed with Dutch plants and (2) for Dutch caterpillars compared with Belgian ones (Fig. 3a). Additionally, Belgian caterpillars fed with Belgian plants and Dutch caterpillars fed with Dutch plants had a similar survival rate (χ^2^ = 0.15; *P* = 0.6998), but Dutch caterpillars fed with Belgian plants showed a higher survival than those fed with Dutch plants (χ^2^ = 12.98; *P* = 0.0003; Fig. 3a). None of the Belgian caterpillars fed with Dutch plants survived.

### Experiment II: effects of plant origin and larval instar on Belgian caterpillars

The 55 Belgian caterpillars were homogeneously distributed among the food treatments according to their initial instar (χ^2^ = 0.23, *P* = 0.63). Caterpillar weight differed only between size groups; caterpillars in the penultimate instar being, as expected, lighter than last instar ones (Caterpillar instar: *F*_1,54_ = 65.49, *P* < 0.0001; mean weight of penultimate instar caterpillars = 34.77 ± 4.07 mg; mean weight of last instar caterpillars = 91.45 ± 19.02 mg).

Growth rate was affected by time, initial instar, food origin, and interaction between food origin and initial instar (see Appendix 1b for model selection and Table 2 for parameter estimates using the best model). Caterpillar growth rate increased with time. It was on average lower for last instar caterpillars than for penultimate instar caterpillars and for caterpillars fed with Dutch plants than for caterpillars fed with Belgian plants. Additionally, when fed with Dutch plants, the growth rate of the caterpillars stabilized or even decreased for penultimate instar and last instar caterpillars, respectively (Fig. 2b).

**Table 2:** Factors affecting caterpillar growth rate (estimated using best model from Appendix 1b)

Parameter	Level	Estimate	Std
Intercept		0.6057	0.1086
Time		0.0971	0.012
Initial instar	Penultimate instar	0.321	0.0943
Food origin	Belgium	0.5135	0.1114
Initial instar*Food origin	Penultimate instar*Belgium	−0.4046	0.1337

For categorical variables, the estimate expresses the difference of the presented with the reference level (fixed to zero). Caterpillar growth rate (1) increased with time, (2) was on average lower for last instar caterpillars, (3) was on average lower for caterpillars fed Dutch plants, (4) stabilized for penultimate instar caterpillars fed Dutch plants, and (5) decreased for last instar caterpillars fed Dutch plants.

Among the 55 caterpillars, 25% reached the (pre-)pupae stage and 75% died. Results of the logistic regression indicated a significant effect of food origin (χ^2^ = 12.11; *P* = 0.0005) and caterpillar instar (*χ*^2^ = 14.94; *P* = 0.0001) on survival rate. On average, higher survival rates were observed (1) for caterpillars fed with Belgian plants (survival: 43%) compared with caterpillars fed with Dutch plants (survival: 8%) and (2) for last instar caterpillars compared with penultimate instar caterpillars (Fig. 3b). There was no interaction effect between food origin and caterpillar instar. Thus, the survival rate was higher for caterpillars fed with Belgian plants in both caterpillar instar groups (Penultimate instar: *χ*^2^ = 4.60; *P* = 0.0319; Last instar: *χ*^2^ = 8.56; *P* = 0.0034; Fig. 3b).

## Discussion

### Better quality of Belgian food plants

Results of this study showed that both Belgian and Dutch caterpillars performed better (i.e. had higher growth and survival rates) when fed with Belgian plants in comparison with Dutch plants. Growth rate differences should be considered cautiously because of a possible developmental plasticity (see example in Roder et al. 2008), but these results are consistent with the results on survival. Survival was on average five times higher when fed with Belgian plants than with Dutch plants, irrespective of caterpillar origin. This implies that Belgian food plants were of overall better quality than Dutch ones.

Changes in host plant quality may arise from changes in the concentration of water, required nutrients, and allelochemicals. As stated by Slansky (1982), responses to such modifications may be inductory (through passive changes in individual's performance) or compensatory (through active changes in behavior). Compensatory feeding in response to lower plant quality was observed for caterpillars of the Monarch butterfly, *Danaus plexippus* and for juvenile *Omocestus viridulus* grasshoppers (Lavoie and Oberhauser 2004; Berner et al. 2005; note that in these two cases, better quality plants were plants with high nitrogen contents). In the velvetbean caterpillars (Slansky and Wheeler 1992), compensatory feeding in response to a reduced nutrient level lowered survival and growth due to the toxicity of an excess of allelochemic consumption. Allelochemicals may act as toxins or reduce the digestibility of plant materials (Mattson 1980). As compensatory feeding was not observed in this study (C. Turlure, pers. obs.), the responses of *B. aquilonaris* caterpillar to changes in host plant quality can be considered as inductory only (i.e. altered growth and survival). Altered performance on lower quality host plants was also observed in other herbivorous insects (see an example in De Bruyn et al. 2002 or a summary in Table 1 of Slansky 1982).

Changes in plant quality are induced by diurnal, seasonal, and ontogenetic cycles of the plant, as well as by environmental changes (Mattson 1980). In this study, several factors may explain the observed difference in host plant quality, i.e. climate, water table height, and nitrogen deposition. First, as the growing season starts approximately 2 weeks earlier at the Dutch lowland site than at the more elevated (and hence with cooler climatic conditions) Belgian site, the Dutch leaves of *V. oxycoccos* were older than the Belgian ones. The chemical content of the plants can change over time due to the circulation of chemicals between shoots and roots or production of plant defensive compounds, plus the water content tends to decrease in older plants (Scriber and Slansky 1981). Hence, the nutritional quality for the caterpillars may be lower in the leaves of Dutch plants used for the rearing. Secondly, the availability of the groundwater (through a higher water table) is greater at the Belgian sites. This is confirmed by field observations (i.e. the luxurious growth of *Menyanthes trifoliata,* which is absent at the Dutch site) and by water samples collected at all sites showing that the availability of minerals in the surface water, for example, Ca and Mg, was much higher in the Belgian sites than in the Dutch site (M. Meijrink, unpubl. data). Hence, the lower water table at the Dutch sites may have reduced plant quality not only through lowered mineral availability but also through drought stress (Mattson 1980). Indeed, *V. oxycoccos* plants develop on *Sphagnum* moss and because of their shallow roots, they rely solely on the water conducted by *Sphagnum* moss (Malmer et al. 1994). Thirdly, nitrogen deposition at the Belgian sites was approximately 26% lower during the last decade (http://www.emep.int), inducing even a greater imbalance in the ratio of nitrogen to other nutrients or a higher content of non-protein compounds in which excess nitrogen is stored in the Dutch host plants (Nijssen and Siepel 2010; Jirousek et al. 2011).

Both the warmer climate, the reduced influence of the groundwater and the higher nitrogen deposition in the Netherlands may have decreased the host plant quality for *B. aquilonaris* caterpillars. To unravel the possible causes of the observed differences in plant quality, further experiments should be conducted taking into account plant phenology (i.e. use of plants reared under conditions controlled for temperature, water and nutrients) and simultaneous plant quality analysis. As an example, using a three-generation bioassay Clancy (1992) found that the performance of the budworm, *Choristoneura occidentalis*, was better explained by the ratio between nitrogen and mineral supplements (Zn in this case) than by the nitrogen concentration in food only.

Regardless of the cause of the lower host plant quality in the Netherlands compared with Belgium, this lower quality implies that Dutch sites fail to comply with one of the four necessary requirements for relocation (see Introduction). Knowing the causes of reduced plant quality shall identify the main ways and means to improve the habitat quality, and experimental trials should be prioritized to test their effectiveness. As shown by Schultz (2001) on a restoration trial for the butterfly *Icaricia icarioides fenderi*, care should be taken to apply the restoration measures on a large scale, as different restoration sites may react differently to the same restoration measures.

### Caterpillars' adaptation to host plant quality

Experiment I showed that the survival rates of caterpillars fed with the plants from their countries of origin were similar. But, one striking result is the increased survival rate of Dutch caterpillars up to 67% when fed with Belgian plants, whereas none of the Belgian caterpillars reached the (pre-) pupae stage when fed with Dutch plants. This suggests that Dutch caterpillars, usually feeding on lower host plant quality, can perform on both lower and higher quality host plants, whereas Belgian caterpillars, usually feeding on high quality plants, cannot switch to lower plant quality. This pattern can be considered as an environmentally induced plastic response of the caterpillars and/or a local adaptation (either through mechanism: phenotypic plasticity or genetic assimilation) to plant quality (West-Eberhard 1989; Meyers and Bull 2002; Pigliucci 2005; Moczek 2010). Belgian populations may lack the so called “key innovations” allowing the maintenance of individual performance on plants with variable quality (Scriber 2010).

For conservation practice, this could prevent relocation of Belgian individuals into Dutch sites. Nevertheless, a possibility to save this species via relocation still exists if adaptation of Belgian caterpillars to low plant quality could be achieved by rearing experiments. Indeed, a previous study demonstrated the ability of codling moth to adapt to changing temperature treatments within one life cycle (Chidawanyika and Terblanche 2011). How fast *B. aquilonaris* caterpillars can adapt to feeding on host plants of poorer quality remains to be tested. Alternatively, other sources of relocations may be used, for example, Danish or Estonian populations, which could better match with host plant quality of the Dutch situation. Moreover, usage of multiple sources is suggested as a preferred option for small populations (Weeks et al. 2011) like those of *B. aquilonaris* in the Netherlands, as this will facilitate the adaptive potential of the newly created mixed population via decrease in the expression of deleterious genes.

The difference in performance between Dutch and Belgian caterpillars on Dutch host plants was, however, not found for the growth rate. One may argue that these results (1) are based on small sample sizes (39 caterpillars in four treatments) and (2) could be biased by the heterogeneous distribution of the caterpillar instars in the four treatments. First, given that this species is of conservation concern, it was the best trade-off between the cost of “loosing” individuals (especially impacting the small Dutch population) versus a full experimental design allowing perfect statistical analysis. Yet, considering Belgian caterpillars, Experiment II gave similar results regarding survival (i.e. lower survival when fed with Dutch plants). Second, the proportion of penultimate instar caterpillars (i.e. the instar with a lower survival as shown in Experiment II) was higher for the Belgian plant × Belgian caterpillar treatment (80%) and similar for the two treatments with Dutch food (55%). Yet, the survival rate was better in with Belgian caterpillars fed Belgian plants, and survival rates differed between Belgian and Dutch caterpillars fed with Dutch plants. For these reasons, we are confident with the above-mentioned conclusions.

Environmentally induced plastic responses and adaptations to local environmental conditions have also been exemplified in other insect species. In the butterfly *Lycaena hippothoe*, Fischer and Fiedler (2002) demonstrated that the changes in development time and number of generations was an adaptation to the local climatic conditions differing according to geographic regions. Zvereva et al. (2010) observed that the level of adaptation to salicylic glycosides concentration in host plants differed between populations of the leaf beetle *Chrysomela lapponica*. Indeed, some populations were either adapted to low, high, or both types of concentration, indicating that such adaptations reflect environmental variations at local scale. Friberg and Wiklund (2010) showed that the influence of the host plant type and the temperature conditions on the decision to enter diapauses differed between the two closely related butterfly species *Lepidea sinapis* and *L. reali*, with an “adaptive developmental phenotypic plasticity” in the first species.

### Implications for conservation scenarios based on relocations

The implications of this study for the conservation of *B. aquilonaris* are twofold. First, we underline the need to improve habitat quality (i.e. the quality of host plants) at the Dutch sites, as it is a likely factor causing species decline. The main differences between the two study regions include the cooler climate, greater groundwater influence, and lower nitrogen deposition in S-Belgium. Which chemical compounds or (im)balances in the *V. oxycoccos* plants determine the higher nutritional value at the Belgian site remains to be tested.

Second, we stress the role of local adaptation in selecting source populations for relocations and assisted migration (Thomas 2011). Indeed, the usual assumption that relocated individuals would contribute in the same way to the population persistence is contradicted here, and this may preclude the use of Belgian individuals in strengthening Dutch populations. The mechanisms actually driving the differential growth and survival of Belgian and Dutch caterpillars on *V. oxycoccos* from different geographic origins remains to be established. However, the ensuing differences in growth and survival response of caterpillars emphasize caution in using populations from different origins in conservation programs. To minimize the risk, the use of multiple source populations may be the best management option. More generally, our work demonstrates that local adaptation is an important factor to consider when selecting source populations for relocation. Its importance may increase in programs of assisted migration in relation to climate change (see Thomas 2011; Moir et al. 2012), because these are more likely to involve environmental differences, like different growing conditions of host plants or even shifts to different host species as recently shown in a butterfly (Pateman et al. 2012). Thus, we recommend testing individuals' performance under the novel environment, prior to the actual release.

## Appendix 1

Modeling caterpillar growth rate according to (a) time, food origin, caterpillar origin, and interaction between food origin and caterpillar origin (Experiment I) and (b) time, initial instar, food origin, and interaction between initial instar and food origin (Experiment II), using generalized models with AICc model selection. For each model, the following information is presented: the list of variables considered, the number of estimated parameters, the AICc value and the difference (Δ) of AICc with the lowest-AICc model. Supported and selected model is the one with the lowest AICc value, i.e. in these cases, the full models marked with ***.

**Table:**

	Experience	Model	Number of parameters	AICc	ΔAICc
(a)	I	Time + Food origin + Caterpillar origin + Interaction ***	5	77.67	0
		Time + Food origin	3	80.46	2.79
		Time + Food origin + Caterpillar origin	4	82.42	4.75
		Time	1	98.07	20.4
		Time + Caterpillar origin	3	99.79	22.12
		Food origin + Caterpillar origin + Interaction	4	132.48	54.81
		Food origin	2	133.15	55.48
		Food origin + Caterpillar origin	3	135.13	57.46
		Intercept only	1	145.08	67.41
		Caterpillar origin	2	146.88	69.21
(b)	II	Time + Food origin + Initial instar + Interaction ***	5	267.62	0
		Time + Food origin + Initial instar	4	274.45	6.83
		Time + Food origin	3	275.42	7.8
		Time + Initial instar	3	285.59	17.97
		Time	1	286.27	18.65
		Food origin + Initial instar + Interaction	4	322.76	55.14
		Food origin + Initial instar	3	327.48	59.86
		Food origin	2	327.55	59.93
		Intercept only	1	335.59	67.97
		Initial instar	2	335.69	68.07
